# Research on Total Ionizing Dose Effect and Reinforcement of SOI-TFET

**DOI:** 10.3390/mi12101232

**Published:** 2021-10-10

**Authors:** Chen Chong, Hongxia Liu, Shulong Wang, Xiaocong Wu

**Affiliations:** Key Laboratory for Wide-Band Gap Semiconductor Materials and Devices of Education, The School of Microlectronics, Xidian University, Xi’an 710071, China; 18829029042@163.com (C.C.); m13720759895@163.com (X.W.)

**Keywords:** silicon-on-insulator (SOI), total ionizing dose (TID), tunneling field effect transistor (TFET)

## Abstract

Since the oxide/source overlap structure can improve the tunneling probability and on-state current of tunneling field effect transistor (TFET) devices, and the silicon-on-insulator (SOI) structure has the effect of resisting the single event effect, SOI-TFET with the oxide/source overlap structure is a device with development potential. The total ionizing dose (TID) effect on SOI-TFET was studied by discussing the switching ratio, band–band tunneling rate, threshold voltage, sub-threshold swing and bipolar effect of the device under different doses of irradiation. At the same time, simulations prove that selecting the proper thickness of the buried oxide (BOX) layer can effectively reduce the influence of the TID effect. This provides a way of direction and method for research on the irradiation effects on the device in the future.

## 1. Introduction

**Figure 1:**
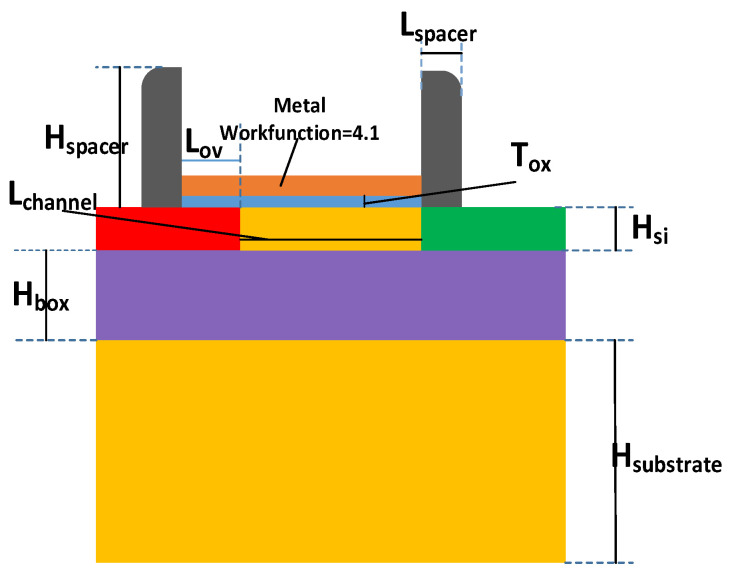
Cross-sectional view of SOI-TFET.

With the development of integrated circuits, the characteristic size of devices is decreasing. TFET has become one of the potential new devices in the field of low voltage and low power consumption circuits because of its low subthreshold swing, high current switching ratio and low subthreshold leakage [1,2,3,4,5]. However, there are few studies on the radiation resistance of TFET devices [6,7,8,9]. This paper focuses on the TID effect in SOI-TFET with oxide/source overlap, the structure of the device is described in Figure 1. The gate of the device overlaps the oxide and the source can increase the on-state current [10,11,12], while the SOI structure can eliminate the latch-up effect and reduce parasitic capacitance [13,14,15,16]. It is found that the current switch ratio and subthreshold swing of the device deteriorates while threshold voltage has a slight drop, but it is not too sensitive after irradiation. The bipolar effect of TFET will be repressed to some degree by radiation, however, the greater the negative voltage of gate, the less the inhibition of radiation on bipolar effect. In addition, the optimal thickness of the buried oxide (BOX) layer is either 20 or 120 nm, where the device has the strongest resistance to the total ionizing dose effects.

## 2. Analysis of Total Dose Effect Mechanism

### 2.1. The Generation of Trapped Charge in the Oxide Layer

When some oxide layers in the device, such as the gate oxide layer, the buried oxide layer of the SOI structure, and the field oxygen isolation layer, are irradiated, a large number of electron-hole pairs will be generated inside. When there is an electric field inside the oxide layer, electrons and the hole will move in the opposite direction under the action of the electric field. Since the mobility of electrons is much greater than that of the holes, the electrons will be swept out of the oxide layer in the order of ps. The formula for the total amount of holes generated by irradiation in the oxide layer is as Equation (1):

(1) $$N_{h}=f\left(E_{ox}\right)g_{0}t_{ox}D$$

where E_ox_ is the electric field intensity in the oxide layer, and g_0_ is the density of electron-hole pairs generated under a unit irradiation dose, which is a material-related parameter. Generally speaking, the average energy required to generate a pair of electron-hole pairs is 3 times the material forbidden bandwidth. t_ox_ is the thickness of the oxide layer, and D is the radiation dose, for which the unit is generally rad (material).

### 2.2. The Generation of Trapped Charge in the Interface State

In addition to introducing trapped charges in the oxide layer, irradiation also introduces interface states at the Si/SiO_2_ interface. At the Si/SiO_2_ interface in the semiconductor, although the interface still maintains the continuity of the crystal lattice, the surface tension of the Si-H and Si-O bonds are very high due to the different lattice constants of the two materials. Irradiation will catalyze the rupture of these bonds, leading to the destruction of the periodic potential field of the lattice at the interface. The destruction of the periodic potential field can be equivalent to superimposing a new fixed potential field on the original periodic potential field. The energy level of the charged center of this electrostatic field is located in the center of the band gap, which is the interface state.

## 3. Methods

### 3.1. Physical Model

The Sentaurus software used in this paper is a computer-aided simulation tool specially under the Synopsys company for device simulation. First, make the device structure in the SDE module, and then add the required physical model in the Sdevice module. For TFET devices, the source region is heavily doped and the condition of E − E_F_ >> k_0_T cannot be met, so the Fermi distribution is adopted. The activation statement in Sdevice is: Physics {Fermi}. In TFET devices, mobility requires additional consideration of ionized impurity scattering models ($\mu_{dop}$), interface scattering models ($\mu_{I\mathrm{nter}Sc}$), and high field velocity saturation models ($\mu_{F}$). The Shockley–Read–Hall (SRH) composite in indirect composite mode is the main composite method used in TFET devices. Moreover, the presence of interface states at the interface of the semiconductor will introduce additional recombination effects, so the surface SRH recombination model needs to be considered. The non-local band-to-band (BTBT) model treats the electric field at each point in the tunnel path as a variable, which is more realistic. Therefore, the non-local BTBT model was adopted.

In this paper, the influence of TID radiation effect on SOI-FDSOI was studied by adding the radiation and interface trap models. The way to add the radiation model is to add the following statement in the physics module of Sdevice:

Radiation (DoseRate = @DoseRate@ DoseTime = (@initialTime@,@finalTime@) DoseTSigma = @DoseTSigma@)

The unit of DoseRate is rad/s, which represents the dose rate of the irradiated device; DoseTime specifies the time range during which the device is exposed to a certain dose rate environment; DoseTSigma (unit: s): can be combined with DoseTime to specify the Gauss of the radiation exposure and the standard deviation of the rise and fall. The generation of electron-hole pairs due to radiation is an electric field-dependent process and is modeled as follows:

(2) $$G_{r}=g_{0}DY\left(E\right)$$

(3) $$Y\left(E\right)={\left(\frac{E+E_{0}}{E+E_{1}}\right)}^{m}$$

where the default value of g_0_ is 7.6 × 10^12^ rad^−1^·cm^−3^, which represents the number of electron-hole pairs generated after a unit volume of the target is irradiated with a dose of 1 rad, D is the radiation dose, Y(E) is the electric field-related hole generation rate function, G_r_ is the number of holes that have not undergone initial recombination, E is the electric field parameter in V/cm, and E_0_, E_1_ and m are constants with sizes of 0.1 V/cm, 1.35 × 10^6^ V/cm, and 0.9, respectively.

### 3.2. Device Model

The device model built in Sentaurus (computer-aided simulation software) is shown in Figure 1, and the model parameters of the device are shown in Table 1. The substrate, source, drain and channel are all made of silicon. BOX is SiO_2_, and the gate oxide layer uses high-K gate dielectric HfO_2_. The side wall is Si_3_N_4_.

## 4. Results and Discussion

In order to study the influence of the TID effect on the performance parameters of the device, this section adopts the method of adding a fixed charge for simulation analysis. The idea of the simulation is to quantify the effect of fixed charge and interface state trap charge caused by irradiation in the BOX layer on the performance of the device, which is equivalent to the density of interface state trap charge. This is achieved by adding the following statement in the command file of Sdevice:

Physics (RegionInterface = “R.box/R.channel”)

{Charge (Uniform Conc=@Conc@e12)}

**Figure 2:**
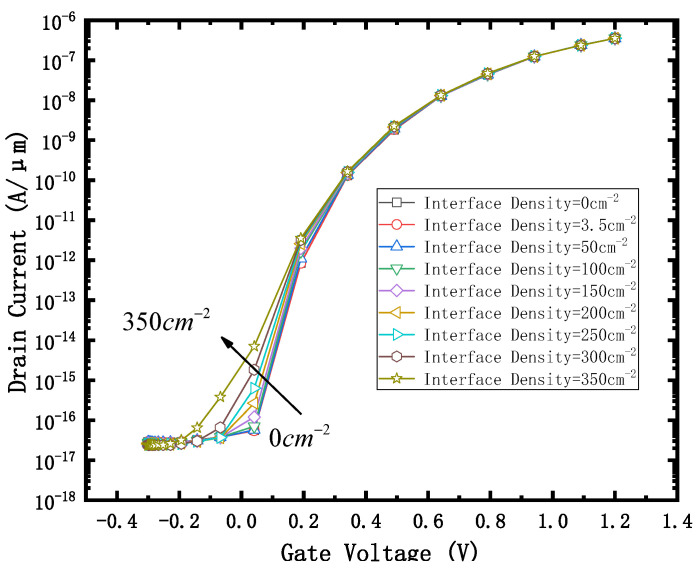
Id-Vg curve with different Interface Density. V_drain_ = 0.8 V.

The effect of positive charge with interface density of 2.93 × 10^12^ cm^−2^ on the device is equivalent to that of a 300 krad (SiO2) dose γ-ray radiating device, and the effect of a positive fixed charge with interface density of 3.26 × 10^12^ cm^−2^ is equivalent to that of 500 krad (SiO_2_) dose γ-ray radiating device, as described in some papers [17,18]. The interface densities of 0, 3.5 × 10^10^, 50 × 10^10^, 100 × 10^10^, 150 × 10^10^, 200 × 10^10^, 250 × 10^10^, 300 × 10^10^and 350 × 10^10^ cm^−2^ were selected to characterize the damage to the device irradiated by 0–500 krad (SiO_2_). The transfer characteristic curve obtained is shown in Figure 2. As the interface charge density increases, the curve shifts to the left, which means that the threshold voltage decreases. The reason is that as the positive charge density on the surface of the BOX is higher, the negative charge density of the channel (near the BOX) is higher, and the device is more likely to be turned on.

### 4.1. Current Switching Ratio I_on_/I_off_ and eBTBTGeneration

**Figure 3:**
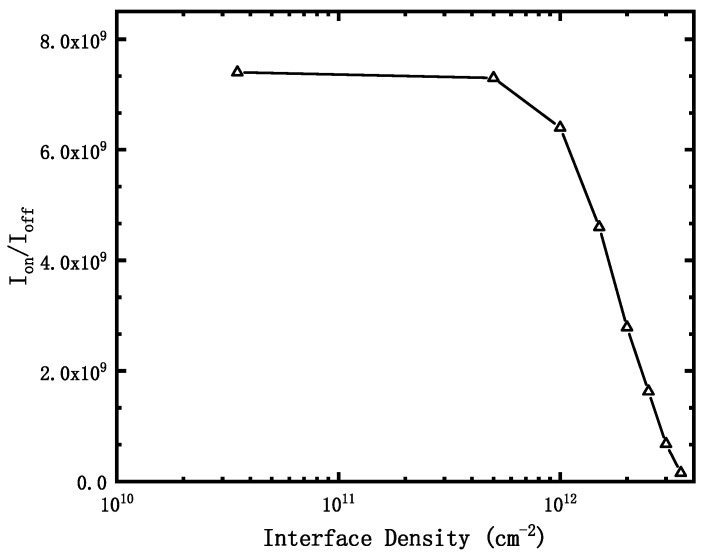
I_on_/I_off_ with different interface density.

Figure 2 shows that when the gate voltage is 1.2 V, the on-state current of the device does not change significantly with the increase of the irradiation dose, while when the gate voltage is 0.2 V, the leakage current of the device increases significantly with the increase of irradiation dose. The I_on_ /I_off_ curve obtained is shown in Figure 3.

It can be seen that when the effect of irradiation on the device is equivalent to a positive charge of 0.5 × 10^12^ cm^−2^ at the interface, the current switching ratio I_on_/I_off_ of the device begins to decrease significantly. When the irradiation dose is 500 krad (SiO_2_) (corresponding to the positive charge density at the interface is about 3.5 × 10^12^ cm^−2^), the I_on_/ I_off_ decreases to 1.56 × 10^8^.

**Figure 4:**
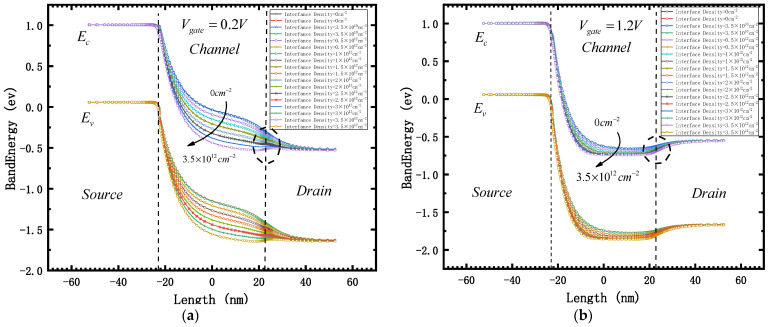
Band diagram with different interface density. (**a**) Gate voltage = 0.2 V; (**b**) gate voltage = 1.2 V.

Compare the energy band diagrams at 0.2 V and 1.2 V (the tangent position is 2 nm upward at the interface between box layer and silicon film), as shown in Figure 4. It is found that:

(1) The smaller the gate voltage, the smaller the control ability of the gate to the channel and barrier region of the device, and the more obvious the influence of the electric field generated by the interface state on the channel and barrier region. Therefore, when the gate voltage is 0.2 V, the change range of the energy band is significantly higher than when the gate voltage is 1.2 V.

(2) The influence of interface states on the energy bands of the channel and barrier regions is much greater than that of source and drain regions. Therefore, the energy bands of source and drain regions do not change obviously, regardless of whether the gate voltage is 0.2 V or 1.2 V.

(3) The higher the concentration of interface state positive charge (the greater the total irradiation dose), the lower the energy band in the channel region. Therefore, when the gate voltage is 0.2 V, the device is at the off state. However, due to the irradiation, the channel energy band is reduced, the barrier difference △Φ between the two ends of the tunneling junction is increased, and the barrier width λ is decreased. These all result in increases to tunneling probability and the leakage current I_off_.

(4) When the gate voltage is 1.2 V, the energy band in the channel region of the device is already very low due to the influence of the gate voltage. Therefore, with the increase of the irradiation dose, although the energy band in the channel region is further reduced, combined with the analysis of (2), it can be found that the influence of the barrier region (the circle part in the two figures) on the electron motion changes from the acceleration of electron drift when the gate voltage is 0.2 V to barrier blocking when is 1.2 V. Combined with the analysis of (1), it can be explained that when the gate voltage is 1.2 V, the on-state current ion of the device does not change greatly.

(5) Combined with the analysis of (3) and (4), we can explain why the current switching ratio I_on_/I_off_ of the device decreases with the increase of irradiation dose.

**Figure 5:**
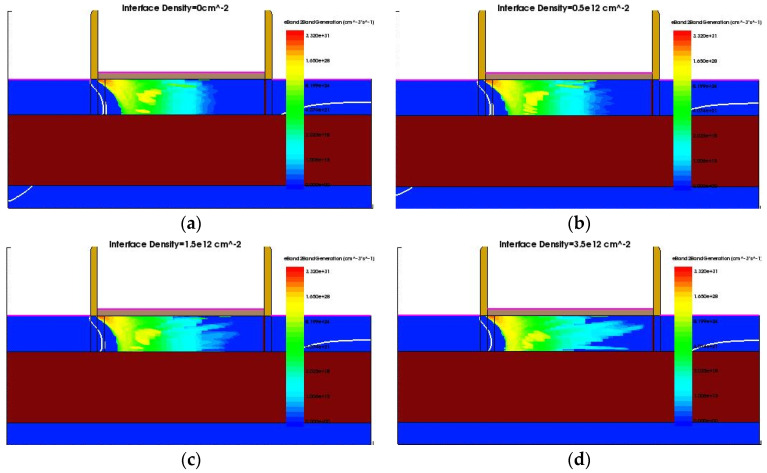
eBTBTGeneration profile. (**a**) Interface density = 0 cm^−2^; (**b**) interface density = 0.5 × 10^12^ cm^−2^; (**c**) interface density = 1.5 × 10^12^ cm^−^^2^; (**d**) interface density = 3.5 × 10^12^ cm^−^^2^.

The electron band-to-band tunneling probability (eBTBTGeneration) of the device (V_gate_ = 0.2 V) with different interface density of states is shown in Figure 5:

It can be seen that, at the off state, the tunneling probability of the tunneling junction is indeed increasing, and the tunneling leakage current is positively correlated with the total radiation dose.

### 4.2. Threshold Voltage and Subthreshold Swing

**Figure 6:**
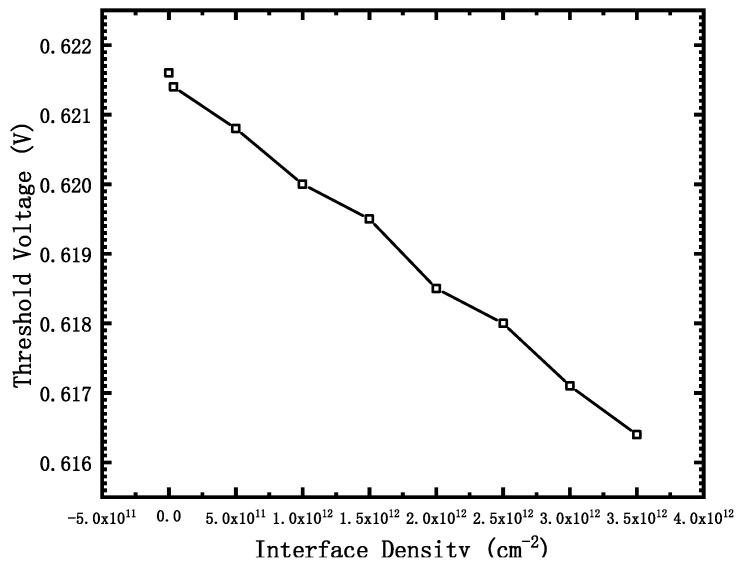
Threshold with different interface density.

Taking the corresponding gate voltage when the current reaches 10^−8^ A/μm as the threshold voltage Vth, the variation curve of the threshold voltage with the dose is shown in Figure 6.

It can be seen that the threshold voltage decreases with the increase of the irradiation dose. The threshold voltage can be understood as the gate voltage applied by the gate when the tunneling current reaches 10^−8^ A/μm. We can know that the positive charge of the interface state will decrease the energy band of the channel region of the device, which is the same as the effect of gate voltage on the energy band in the channel region. Therefore, irradiation will take a portion of the gate voltage, making the device easier to turn on. However, the threshold voltage of the device is not seriously affected by the TID effects.

For TFET devices, the average subthreshold swing is generally used to measure the subthreshold swing characteristics, the formula is as follows Equation (4):

(4) $$SS_{ave}=\frac{V_{g2}-V_{g1}}{\log_{10}I_{2}-\log_{10}I_{1}}$$

**Figure 7:**
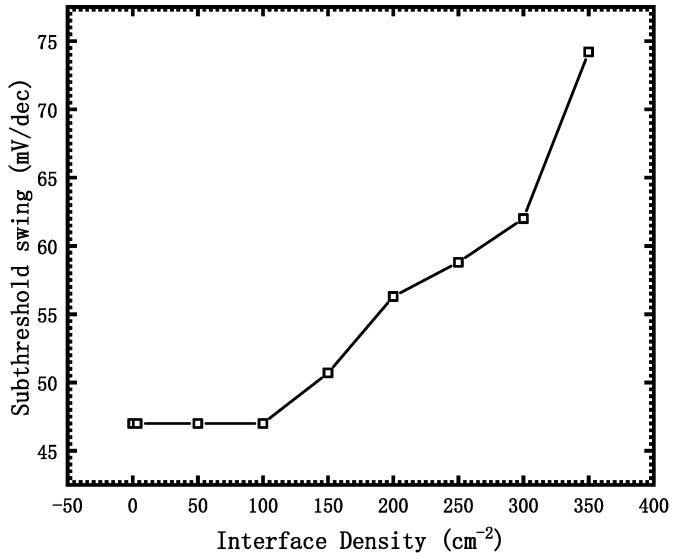
Subthreshold swing with different Interface Density.

The curve of average subthreshold swing extracted from the transfer characteristic curve (the gate voltage corresponding to leakage current of 10^−15^ magnitude is V_g1_, and the gate voltage corresponding to leakage current of 10^−10^ magnitude is V_g2_) with the dose is shown in Figure 7.

It can be seen that the subthreshold swing of the device begins to increase obviously after the interface density of states reaches 100 cm^−2^. After the interface density of states reaches 350 cm^−2^, the subthreshold swing of the device deteriorates from 47 mV/dec to 74.2 mV/dec.

From the definition of subthreshold swing, the derivation of Equation (5) can be achieved:

(5) $$SS=\frac{dV_{G}}{d\log_{10}I_{D}}=\frac{dV_{G}}{d\psi_{s}}\cdot \frac{d\psi_{s}}{d\log_{10}I_{D}}=(1+\frac{C_{D}}{C_{ox}})\cdot \frac{d\psi_{s}}{d\log_{10}I_{D}}$$

where C_ox_ is the gate oxide capacitance, C_D_ is the depletion capacitance and ψ_s_ is the surface potential. Due to the existence of the interface trap charge, a trap capacitance in parallel with C_D_ will be added, which will increase the equivalent depletion layer capacitance C_D’_. The higher the interface trap concentration, the larger the equivalent depletion layer capacitance C_D’_, and the larger the subthreshold swing.

### 4.3. Bipolar Effect

**Figure 8:**
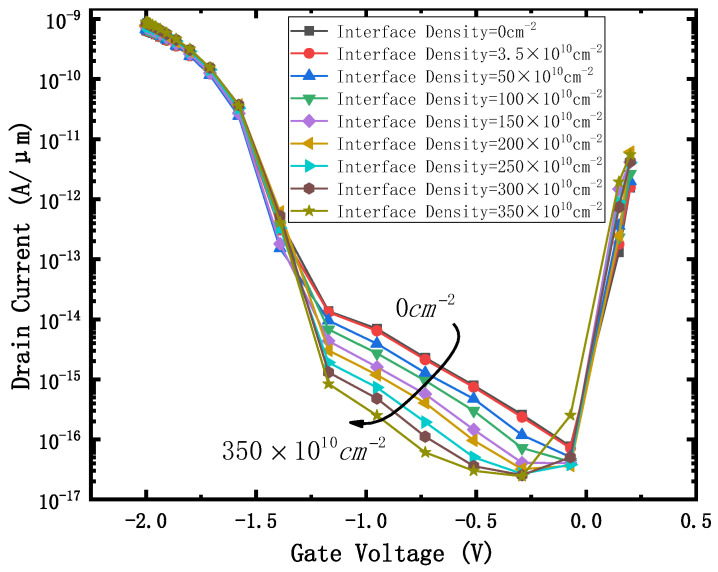
I_d_-negative gate voltage with different Interface Density.

Because of the symmetrical structure of source and drain in TFET, taking n-type TFET as an example, when a large negative pressure is applied to the gate, band to band tunneling will occur in the drain region and body region, resulting in the increase of leakage current and the bipolar effect [19,20,21,22], as seen in Figure 8.

It can be seen that when the absolute value of negative voltage is very low (−1.2 V~0 V), the conduction current of the device decreases with the increase of irradiation dose; when the absolute value of negative voltage is large (>1.2 V), and the irradiation dose has little effect on the conduction current of the device.

**Figure 9:**
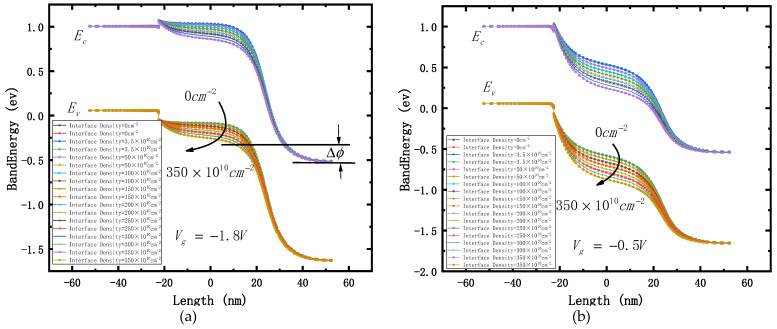
Band diagram with different interface density. (**a**) Gate voltage = −1.8 V; (**b**) gate voltage = −0.5 V.

The tangent diagram of the energy band of the device under different negative gate voltages is shown in Figure 9 (transverse tangent of 2 nm upward at the interface between box layer and silicon film). It can be seen that the higher the irradiation dose, the lower the energy band in the channel region of the device. The more negative the gate voltage, the higher the energy band in the channel region, which indicates that the polarity of the electric field generated by the gate voltage in the channel region of the device is opposite to that caused by the total dose of irradiation. When the negative bias voltage is greater than −1.2 V, the electric field generated by the gate voltage is equivalent to the additional electric field caused by the total dose irradiation, and the total dose effect is obvious. It can be seen in Figure 9b that the higher the irradiation dose, the lower the channel energy band, and the more difficult it is to open the tunneling junction. Therefore, the negative conduction current will be suppressed with the increase of the irradiation dose. When the negative bias voltage is less than −1.2 V, the electric field intensity generated by the gate voltage in the channel increases, and the effect of the additional electric field caused by the total dose irradiation is not obvious. It can be seen in Figure 9a that, although the irradiation dose is equivalent to a 350 × 10^10^ cm^−2^ interface-positive charge, the effect of the gate voltage still makes the tunneling junction have a considerable barrier difference ΔΦ, and the effect of the irradiation dose on the negative guide current is not obvious.

## 5. Hardening of BOX layer

**Figure 10:**
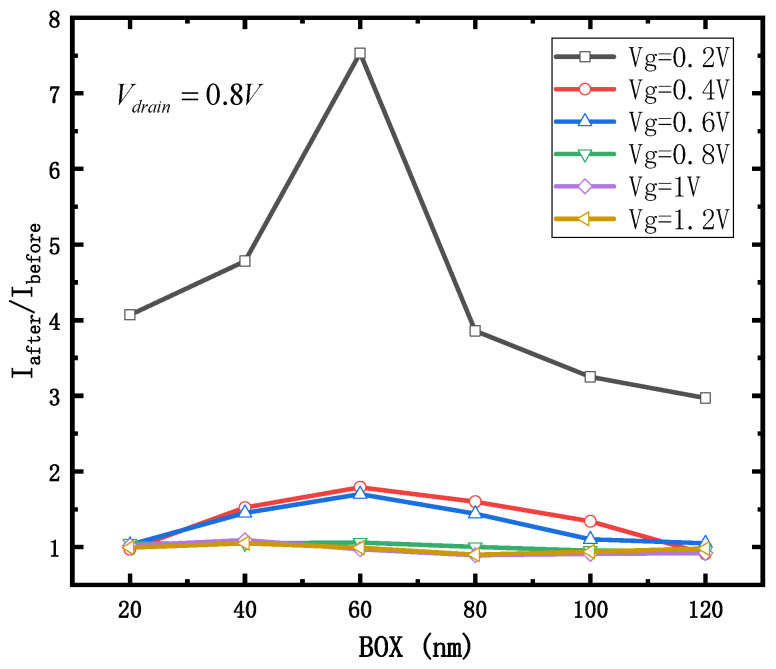
I_after_/I_before_ with different thicknesses of BOX layer.

The influence of a total dose irradiation on the device is quantified by the ratio of the drain current I_after_ after irradiation to the drain current I_before_ when the device is not irradiated. The thicknesses of the simulated box layers were 20, 40, 60, 80, 100 and 120 nm, respectively. The drain bias of the device was 0.8V, the gate voltage of the device was scanned from 0.2 V to 1.2 V, and the scanning point interval was 0.2 V. The simulation method was to add the radiation model and the traps model. The irradiation rate was 100 rad/s and the irradiation time was (0 s, 2000 s). The curves of I_after_/I_before_ with different thicknesses of the box layer are shown in Figure 10:

It can be seen that, with the increase of the thickness of the box layer, the ratio of I_after_/I_before_ first increases, and then decreases. Generally speaking, when the thickness of the box layer is about 20 nm and 120 nm, the influence of the box layer on the total dose effect of the device is the smallest.

When the thickness of the box layer is 20 nm, the total dose effect will be at a very low level due to the small volume of the box layer and the limited area of fixed charge induced by irradiation.

**Figure 11:**
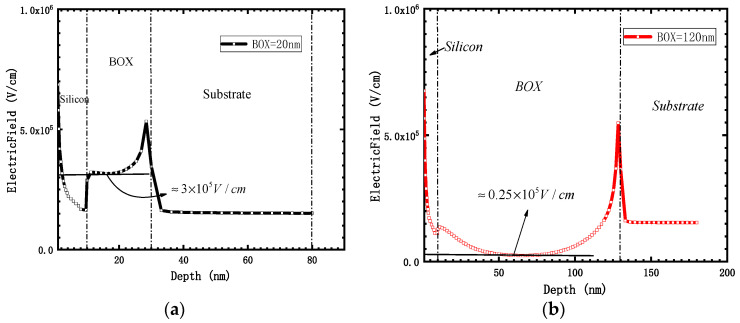
Tangent diagram of vertical distribution of electric field, (**a**) BOX = 20 nm; (**b**) BOX = 120 nm.

When the thickness of the box layer is 120 nm, although the volume of the box layer of the device is increased under the same bias, the thicker the box layer, the smaller the internal electric field intensity, which is positively correlated with the generation rate of holes per unit volume. As shown in Figure 11 (V_gate_ = 1.2 V, along the central vertical tangent of the channel), it can be seen that when the box layer thickness was 20 nm, most of the field strength of the device box layer was maintained at 3 × 10^5^ V/cm. When the box layer thickness was 120 nm, most of the field strength of the device box layer was maintained at 0.25 × 10^5^ V/cm, which is reduced by 12 times.

## 6. Conclusions

This paper focuses on the TID effect in SOI-TFET with an oxide/source overlap. It is found that radiation will obviously deteriorate the subthreshold swing and current switch ratio of device, while the threshold voltage is not affected obviously. When the negative voltage is not too large, the bipolar effect will be suppressed to some degree. The optimal thickness of the BOX layer is either 20 nm or 120 nm, where the device has the strongest resistance to total ionizing dose effects. This is of great significance for studying the radiation effect of TFET devices.

## Figures and Tables

**Table 1:** Device parameters used for the simulation.

Parameter Name	Symbol	Value	Unit
Channel length	L_channel_	45	nm
Gate oxide thickness	T_ox_	2	nm
Silicon film thickness	H_Si_	10	nm
BOX thickness	H_box_	20	nm
Substrate thickness	H_substrate_	50	nm
Metal work function	Φ_M_	4.17	eV
Channel doping	Boron	1 × 10^16^	cm^−^^3^
Substrate doping	Boron	1 × 10^16^	cm^−^^3^
Spacer height	H_spacer_	20	nm
Spacer length	L_spacer_	2	nm
Gate source overlap length	L_ov_	6	nm
